# Comparative Study of MAPP Compatibilization and H_2_O_2_ Surface Treatment for Recycled GFRP-Reinforced Wood–Plastic Composites: Interfacial Properties and Performance

**DOI:** 10.3390/ma19163487

**Published:** 2026-08-18

**Authors:** Tong Wang, Ao Li, Linchong Wei, Hongguang Liu, Bin Luo, Li Li

**Affiliations:** College of Materials Science and Technology, Beijing Forestry University, Beijing 100083, China; wttt0603@outlook.com (T.W.); liaoclxy123@bjfu.edu.cn (A.L.); weilc197@163.com (L.W.); luobincl@bjfu.edu.cn (B.L.); lili-email@263.net (L.L.)

**Keywords:** wood–plastic composites, recycled GFRP, interfacial modification, MAPP, hydrogen peroxide, wind turbine blade recycling, mechanical properties, surface free energy

## Abstract

Recycled glass fiber-reinforced polymer (GFRP) powder from decommissioned wind turbine blades offers sustainable reinforcement for wood–plastic composites (WPCs), but its efficiency is limited by poor interfacial adhesion with the polypropylene (PP) matrix caused by surface epoxy residues. In this study, GFRP/WPCs with a fixed formulation of 15 wt% GFRP, 10 wt% wood flour, and 75 wt% PP were used to compare two modification strategies: MAPP compatibilization (1–7 wt%) and H_2_O_2_ treatment (5–30%). MAPP modification improved the mechanical properties of GFRP/WPCs, with the optimal concentration varying by property: flexural strength reached its maximum (75.45 MPa, +24.7%) at 1 wt% MAPP, while tensile strength (+9.2%), flexural modulus (+20.7%), and impact strength (+18.9%) were maximized at 3 wt% MAPP. H_2_O_2_ at 10% achieved higher strength gains (tensile: +23.65%, i.e., 26.7 MPa; flexural: +27.93%, i.e., 77.4 MPa; impact: +35.29%, i.e., 16.98 kJ/m^2^) but moderately reduced flexural modulus. FTIR confirmed up to 71.7% epoxy removal by H_2_O_2_, exposing cleaner fibers. Both modifications slightly lowered thermal decomposition temperatures but increased char residues and PP crystallinity via enhanced nucleation. SEM showed that MAPP created a compatible interphase, while H_2_O_2_ enabled direct mechanical interlocking. Surface free energy analysis revealed that MAPP increased polar components, whereas H_2_O_2_ increased dispersive components. Overall, MAPP offers simpler processing and balanced properties, while H_2_O_2_ provides superior strength at the cost of some stiffness—providing practical guidance for tailoring recycled GFRP/WPCs for construction and sustainable applications.

## 1. Introduction

The rapid expansion of wind energy has led to an impending wave of wind turbine blade decommissioning, with an estimated 17.6 million tons of blade waste expected in China by 2024 alone [1,2]. Wind turbine blades are primarily composed of glass fiber-reinforced polymer (GFRP) composites, which pose significant environmental challenges at end-of-life due to their crosslinked thermoset matrix and durable glass fibers [3,4]. Mechanical recycling, which involves crushing and sieving blades into powder or short-fiber fractions, offers a low-cost and environmentally friendly approach [5,6]. However, the recycled GFRP (rGFRP) powder typically contains residual epoxy resin adhered to glass fiber surfaces, which compromises its reinforcing efficiency when incorporated into new polymer matrices [7,8,9].

Wood–plastic composites (WPCs), composed of thermoplastics and wood fibers, have gained widespread acceptance in building, automotive, and outdoor applications due to their renewability, low maintenance, and good processability [10,11]. Incorporating rGFRP powder into WPCs represents an attractive valorization route that simultaneously addresses waste management and material performance enhancement. In our previous work [12], we systematically optimized the formulation of rGFRP-reinforced WPCs and determined the optimal composition: 10 wt% poplar wood flour, 15 wt% S-GFRP powder (passing through a 100-mesh sieve, median particle size 25.4 μm), and 75 wt% polypropylene (PP). This formulation achieved balanced mechanical properties (tensile strength 21.57 MPa, flexural strength 59.70 MPa, flexural modulus 2595.12 MPa, impact strength 12.55 kJ/m^2^), meeting the requirements for building formwork applications. However, we also observed that further increasing the GFRP content led to fiber agglomeration and interfacial debonding, and the presence of residual epoxy resin created weak anchor points between the filler and the PP matrix, limiting the full exploitation of the reinforcing potential.

To overcome these interfacial issues, two widely explored approaches are compatibilizer addition and fiber surface treatment. Maleic anhydride-grafted polypropylene (MAPP) is a well-established compatibilizer that enhances interfacial adhesion by forming hydrogen bonds or covalent ester linkages with hydroxyl groups on wood fibers and glass fibers, while its PP backbone provides chain entanglement with the PP matrix [13,14]. The anhydride groups of MAPP can react with surface hydroxyls, reducing the polarity mismatch and promoting uniform filler dispersion [15,16]. However, MAPP cannot eliminate the intrinsic weakness caused by the epoxy layer on the rGFRP surface; the interface is still mediated through this epoxy layer, which remains the weak link [17].

Alternatively, chemical treatments can remove or modify the surface contaminants on rGFRP. Hydrogen peroxide (H_2_O_2_) has been employed as a mild oxidizing agent to degrade organic contaminants, including epoxy resins, under relatively mild conditions (90 °C, atmospheric pressure) [18,19]. H_2_O_2_ treatment can expose cleaner glass fiber surfaces, increasing the contact area and enabling direct fiber–matrix bonding through mechanical interlocking and, potentially, hydrogen bonding with polar groups on wood fibers. The concentration of H_2_O_2_ is a critical parameter: insufficient treatment leaves residual epoxy, while excessive oxidation may damage the glass fibers themselves, reducing their reinforcing efficiency [20,21].

Despite the potential of both approaches, there has been no comprehensive comparative study on MAPP compatibilization versus H_2_O_2_ surface treatment for rGFRP/WPC systems, especially based on a well-defined optimized formulation. Furthermore, the underlying mechanisms by which these modification strategies affect interfacial chemistry, surface energy, crystallization behavior, and overall composite performance have not been fully elucidated. This study aims to fill this knowledge gap by systematically investigating both modification approaches on the previously optimized formulation. Specifically, we (1) optimize the MAPP content (1–7 wt%) in rGFRP/WPCs; (2) optimize the H_2_O_2_ concentration (5–30%) for rGFRP surface treatment; (3) characterize the chemical, thermal, morphological, and surface energy changes after modification; and (4) establish the structure–property relationships linking each modification strategy to composite performance. This work provides fundamental insights into interfacial engineering strategies for sustainable composite materials derived from renewable and recycled sources, with practical implications for the construction and building materials sectors.

## 2. Materials and Methods

During the preparation of this manuscript, the authors used Doubao AI (version 14.6.0) for image enhancement purposes to improve the clarity and readability of the figures. The authors have reviewed and edited the output and take full responsibility for the content of this publication.

### 2.1. Materials

The experimental materials used in this study are listed in Table 1.

### 2.2. Equipment

The equipment required in this study is listed in Table 2.

### 2.3. Experimental Methods

#### 2.3.1. Preparation of MAPP-Modified GFRP/WPCs

A certain amount of MAPP and PP were dried in an oven at 80 °C for 2 h to remove surface moisture. The dried MAPP and PP were thoroughly mixed and then fed into a pressurized internal mixer (ZG-0.5L, Dongguan Zhenggong Electromechanical Equipment Technology Co., Ltd.). The compounding was carried out at 180 °C with a rotor speed of 45 rpm for 2 min. The compounded mixture was then taken out and crushed into small granules. Subsequently, wood flour (WF) and S-GFRP powder were weighed according to Table 3 and mixed with the above granules, and the mixture was compounded for an additional 6 min under the same conditions to ensure uniform dispersion of wood flour and GFRP in the polymer matrix. The compounded blend was crushed again into fine granules. Wood flour content was fixed at 10 wt% for all formulations, as specified in Table 3 and Table 4.

The granules were uniformly placed into a stainless-steel mold and compression-molded using a 100T universal press (BY302X2/2, Suzhou Xinxieli Co., Ltd.) at 180 °C under a pressure of 5 MPa for 5 min. The mold with the specimen was then transferred to a cold press (CGYJ-100, Shijiazhuang Cangao High-frequency Machinery Co., Ltd.) and cooled under pressure at room temperature for 5 min until fully solidified. After demoulding, MAPP-modified GFRP/WPC sheets with a thickness of 4 mm were obtained for subsequent characterization.

#### 2.3.2. Preparation of H_2_O_2_-Modified GFRP/WPC

According to Table 4, 400 mL of H_2_O_2_ solution at the desired concentration was prepared by diluting 30 wt% H_2_O_2_ with deionised water. Then, 100 g of GFRP powder was slowly added to the prepared H_2_O_2_ solution and reacted at 90 °C for 6 h under continuous stirring on a magnetic heating stirrer. The reaction temperature was monitored continuously using a thermocouple immersed in the solution, and the temperature was maintained at 90 ± 2 °C throughout the reaction. The mild heating conditions and the use of a controlled magnetic stirrer ensured that the exothermic nature of H_2_O_2_ decomposition did not cause runaway temperature increases, which is particularly important when treating fine powders with high surface area. After the reaction, the modified GFRP powder was filtered out from the beaker, washed repeatedly with deionized water until neutral, and dried in an oven at 80 °C to constant weight. The degradation degree (Dd) of GFRP was calculated using Equation (1). In addition, the liquid decomposition products (filtrate) were collected and heated at 40 °C for 24 h to remove water for subsequent chemical characterization.

The dried H_2_O_2_-modified GFRP powder was weighed according to Table 4 and mixed with PP and wood flour. The mixture was fed into the internal mixer and compounded at 180 °C with a rotor speed of 45 rpm for 2 min. The compounded blend was taken out and crushed into fine granules. The granules were uniformly placed into a stainless-steel mold and compression-molded using a 100T universal press (BY302X2/2, Suzhou Xinxieli Co., Ltd.) at 180 °C under a pressure of 5 MPa for 5 min, then transferred to a cold press (CGYJ-100, Shijiazhuang Cangao High-frequency Machinery Co., Ltd.) and cooled under pressure at room temperature for 5 min. After demoulding, H_2_O_2_-modified GFRP/WPC sheets with a thickness of 4 mm were obtained.

(1) $$D_{d}=\frac{m_{1}-m_{2}}{m_{1}\times 0.2303}\times 100$$

where $m_{1}$ is the mass of GFRP before degradation (g), $m_{2}$ is the mass of dried GFRP after degradation (g), and 0.2303 is the mass fractions of organic epoxy resin in the pristine GFRP (i.e., 23.03 wt%).

### 2.4. Basic Mechanical Properties of Modified GFRP/WPC

All specimens for tensile, flexural, and impact tests were cut from the same compression-molded sheet for each formulation to ensure consistency and eliminate batch-to-batch variability.

#### 2.4.1. Tensile Properties

Type II specimens were prepared according to the Chinese national standard GB/T 1447-2005 (“Test method for tensile properties of fiber-reinforced plastics”) [22]. The specimen dimensions were 250 mm × 100 mm × 4 mm. Five specimens were taken from each group, and the testing speed was set to 5 mm/min.

#### 2.4.2. Flexural Properties

The flexural properties of GFRP/WPC, including flexural strength (MOR) and flexural modulus (MOE), were measured in accordance with GB/T 1449-2005 (“Test method for flexural properties of fibre-reinforced plastics”) [23]. The standard specimen size was 100 mm × 10 mm × 4 mm, with a support span of 70 mm. The specimens were numbered and m rked, and the width and thickness at any three points within the middle one-third span were measured to calculate the average values. The test was performed using the three-point bending method with a uniform loading speed of 5 mm/min. Five specimens were tested per group.

#### 2.4.3. Impact Strength

Unnotched specimens were prepared according to GB/T 1043-2008 (“Plastics—Determination of charpy impact properties—Part 1: Non-instrumented impact test”) [24]. The specimen size was 80 mm × 10 mm × 4 mm. The energy absorbed by the specimen was required to be within 10–80% of the rated energy of the pendulum hammer. If multiple hammers satisfied this condition, the hammer with the highest rated energy was selected for testing. Five specimens were tested per group.

### 2.5. Micro-Morphological Observation of Modified GFRP/WPC

The impact fracture surfaces of the GFRP/WPCs modified by different methods were observed using field-emission scanning electron microscopy (FE-SEM). Small specimens with a thickness of approximately 2 mm were cut from the central region of the impact-tested bars. Prior to observation, the fracture surfaces were sputter-coated with a thin layer of gold to improve electrical conductivity. The accelerating voltage was set to 3.0 kV, and the fracture morphologies were examined at various magnifications (including ×500) to evaluate the interfacial bonding between the reinforcements (GFRP and wood flour) and the PP matrix.

### 2.6. Surface Free Energy Measurement of Modified GFRP/WPC

The crushed granules obtained after internal mixing were uniformly placed into a stainless-steel mold and compression-molded using a 100T universal press at 180 °C under a pressure of 5 MPa for 5 min. The mold was then transferred to a cold press and cooled under pressure at room temperature for 5 min until fully solidified. After demoulding, GFRP/WPC sheets with a thickness of 4 mm were obtained.

The surface free energy of the modified GFRP/WPCs was measured using an optical contact angle goniometer. The composite specimens were cut into flat, smooth pieces with dimensions of approximately 20 mm × 20 mm × 4 mm and cleaned with ethanol to remove surface contaminants prior to testing. Distilled water and ethylene glycol were used as probe liquids. For each measurement, a 3 µL droplet of the test liquid was deposited onto the specimen surface at a rate of 0.1 µL/ms using an automated syringe. The contact angles were recorded at three different positions on each specimen, and the average values were calculated using the instrument’s built-in software. The surface free energy, along with its polar and dispersive components, was determined according to the Owens–Wendt method using Equations (2) and (3) [25]. The surface free energy and polar component values used for distilled water were 72.8 $mJ/m^{2}$ and 51.0 $mJ/m^{2}$, respectively, and those for ethylene glycol were 48.0 $mJ/m^{2}$ and 19.0 $mJ/m^{2}$, respectively.

(2) $$\gamma_{1}\left(1+\cos\theta \right)=2\times \sqrt{\left(\gamma_{S}^{P}\gamma_{1}^{P}\right)+\left(\gamma_{S}^{h}\gamma_{1}^{h}\right)}$$

(3) $$\gamma_{w}=\gamma_{s}^{p}+\gamma_{s}^{h}$$

where $\gamma_{l}$ is the surface energy of the test liquid, θ is the contact angle between the sample and the test liquid; $\gamma_{l}^{P}$ is the polar component of the surface energy of the test liquid, $\gamma_{l}^{h}$ is the dispersive component of the surface energy of the test liquid; $\gamma_{s}^{P}$ is the polar component of the surface energy of the sample, $\gamma_{s}^{h}$ is the dispersive component of the surface energy of the sample, and $\gamma_{\omega }$ is the surface energy of the sample.

### 2.7. Fourier-Transform Infrared Spectroscopy of Modified GFRP Powder

The GFRP powder samples before and after H_2_O_2_ modification were dried in an oven at 80 °C for 2 h to remove surface moisture. A small amount of the dried powder was thoroughly mixed and ground with spectroscopic-grade KBr, and the mixture was pressed into a transparent pellet with a diameter of approximately 13 mm and a thickness of approximately 1 mm using an infrared press. Fourier-transform infrared spectra were acquired using an FTIR spectrometer (Nicolet iS20, Thermo Scientific, USA) in transmission mode over the wavenumber range of 4000–400 cm^−1^ at a resolution of 4 cm^−1^ with 32 scans per spectrum. A background scan was performed using air as the reference before each sample measurement, and the transmission spectra were recorded.

For the liquid decomposition products, the liquid film method was adopted: a small amount of the liquid product was dropped onto a KBr window and dried under an infrared lamp to form a thin film, and the infrared spectra were then collected using the same instrumental parameters. All samples were tested under identical conditions to minimize environmental interference.

### 2.8. Thermal Properties of Modified GFRP/WPC

#### 2.8.1. Thermogravimetric (TG) Analysis

Thermogravimetric analysis was performed using a thermogravimetric analyzer (HITACHI STA200, Hitachi, Japan) to evaluate the thermal stability of the GFRP/WPCs before and after modification. Approximately 5–10 mg of each sample was placed in an alumina crucible and heated from room temperature to 600 °C at a heating rate of 10 °C/min under a nitrogen atmosphere with a flow rate of 50 mL/min. The mass loss curves (TG) and their first-derivative curves (DTG) were recorded.

#### 2.8.2. Differential Scanning Calorimetry (DSC)

The melting and crystallization behaviors of the samples were measured using a differential scanning calorimeter (DSC200, Hitachi, Japan). Approximately 5–10 mg of each sample was sealed in an aluminum pan and heated from room temperature to 200 °C at a rate of 10 °C/min under a nitrogen atmosphere, and held isothermally at 200 °C for 5 min to eliminate thermal history. The crystallinity values reported in this study were calculated from the second heating scan following the elimination of thermal history. This approach measures the intrinsic crystallinity of the PP matrix after controlled cooling from the melt, reflecting the nucleating effect of the fillers on PP crystallization under identical thermal conditions. The samples were then cooled to room temperature at 10 °C/min to record the cooling curve (crystallization curve), followed by a second heating scan at the same rate to 200 °C to record the melting curve. All measurements were performed under nitrogen protection.

The crystallinity (Χ) of the samples was calculated using Equation (4):

(4) $$Xc=100\%\times \frac{\Delta H_{m}}{\Delta H_{f}\times w}$$

where $∆H_{m}$ is the melting enthalpy of the specimen (J/g), w is the mass fraction of PP in the composite, and $∆H_{f}$ is the melting enthalpy of 100% crystalline isotactic polypropylene, taken as 209 J/g.

## 3. Results and Discussion

### 3.1. Effect of MAPP Modification on Properties of GFRP/WPC

#### 3.1.1. Effect of MAPP Modification on Mechanical Properties of GFRP/WPC

**Figure 1:**
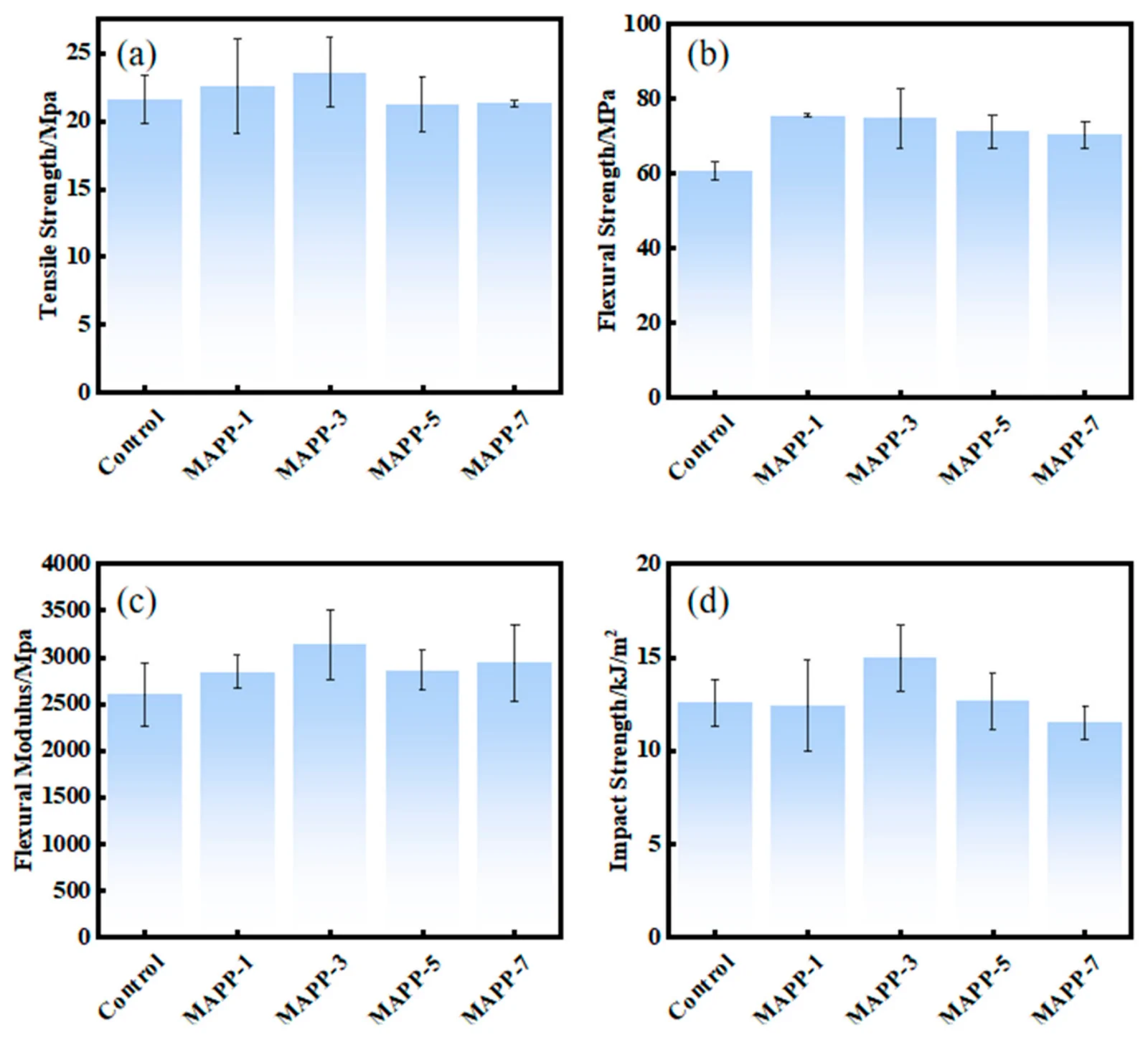
Mechanical Properties of MAPP-Modified GFRP/WPC: (**a**) tensile strength; (**b**) flexural strength; (**c**) flexural modulus; (**d**) impact strength.

Figure 1 shows the mechanical properties of GFRP/WPCs modified with different mass fractions of MAPP. It can be seen that all mechanical properties of the MAPP-modified GFRP/WPC are superior to those of the unmodified control group. As the MAPP content increases, the improvement in mechanical properties declines after exceeding 3%. The tensile strength, flexural modulus and impact strength all reach their maximum values at a MAPP content of 3%, namely 23.6 MPa, 3132.5 MPa and 14.9 kJ/m^2^, which are 9.2%, 20.7% and 18.9% higher than those of the unmodified GFRP/WPC, respectively. The flexural strength reaches its maximum of 75.5 MPa at 1% MAPP, representing a 24.7% increase over the unmodified sample. It should be noted that while the mean flexural strength was highest at 1 wt% MAPP (75.5 MPa), the deviation ranges shown in Figure 1b indicate that the difference between the values at 1 wt% and 3 wt% MAPP may not be statistically significant. Given the scatter in the data, 3 wt% MAPP could be considered practically comparable or even preferable when considering the simultaneous improvements in tensile strength, flexural modulus, and impact strength achieved at this concentration. Therefore, the addition of MAPP has a positive effect on the mechanical properties of GFRP/WPCs.

Compared with the unmodified GFRP/WPCs, the introduction of MAPP greatly enhances the interfacial bonding between wood flour, GFRP and the matrix resin. Since both wood flour and GFRP surfaces contain a large number of hydroxyl groups, MAPP can form hydrogen bonds with the hydroxyl groups on GFRP and wood flour, reducing their polar surface components. At the same time, MAPP reduces the agglomeration tendency between wood flour particles and GFRP powder in the composite, allowing them to disperse more uniformly in the PP matrix and perform more effectively, thus avoiding premature failure due to stress concentration from agglomeration, and further improving the tensile strength, flexural strength and impact strength. Similar improvements in mechanical properties of WPCs through MAPP compatibilization have been reported in the international literature. For instance, Poletto et al. [15] demonstrated that MAPP significantly enhanced the interfacial adhesion between wood flour and recycled polystyrene, leading to improved tensile and flexural properties. Fatima Ezzahrae et al. [16] observed comparable effects in HDPE/wood flour composites, where MAPP addition improved both mechanical performance and thermal stability. The nucleation effect of MAPP on PP crystallization observed in our study is also consistent with findings by Chen et al. [26],who reported increased crystallinity in maleic anhydride-grafted systems. The increase in flexural modulus (+20.7%) can be attributed to two synergistic effects. First, MAPP acts as a compatibilizing agent that enhances stress transfer across the interface through covalent and hydrogen bonding between the anhydride groups of MAPP and the hydroxyl groups on the wood flour and glass fiber surfaces, while its PP backbone provides chain entanglement with the PP matrix. This improved interfacial adhesion enables more effective load transfer from the matrix to the rigid fillers, thereby increasing the composite’s resistance to bending deformation. Second, the nucleation effect of MAPP promotes the crystallinity of the PP matrix, and the increased crystallinity contributes to enhanced stiffness. The higher melt flow index of MAPP (30 g/10 min) compared to PP (3.14 g/10 min) may facilitate filler dispersion during compounding, but this effect alone does not explain the modulus increase; rather, it is the combination of improved interfacial adhesion and enhanced matrix crystallinity that drives the observed improvement in flexural modulus.

Nevertheless, Figure 1 also shows that when the MAPP content exceeds a certain level, the mechanical properties decrease to varying degrees. This is because excess MAPP cannot effectively bond with GFRP and wood flour. The remaining MAPP exists as free species between the matrix resin and the reinforcements, forming defects. Under external stress, cracks easily initiate and propagate in this weak interfacial layer, leading to premature failure at lower stress levels. This phenomenon is especially obvious in the impact strength variation shown in Figure 1d.

This decrease can be attributed to the embrittlement of the interface at high MAPP content. While moderate MAPP addition improves interfacial adhesion and energy absorption, excess MAPP tends to form a weak boundary layer that promotes brittle fracture, reducing impact resistance. This trade-off between interfacial enhancement and embrittlement is common in compatibilized polymer blends and should be considered when optimizing MAPP content.

To validate the statistical significance of the observed mechanical property improvements, one-way analysis of variance (ANOVA) followed by Tukey’s HSD post hoc test was performed. The results confirmed that the improvements in tensile strength, flexural modulus, and impact strength at 3 wt% MAPP were statistically significant (*p* < 0.05) compared to the control. However, the difference in flexural strength between 1 wt% and 3 wt% MAPP was not statistically significant (*p* > 0.05), supporting the view that 3 wt% MAPP is practically comparable or preferable when considering the simultaneous improvements in other properties.

Overall, the improvement in mechanical properties of GFRP/WPC by MAPP can be attributed to two main effects. One is the “bridge” role of MAPP, which enhances the interfacial adhesion between wood flour, GFRP and the matrix resin. The second is the soft and processable nature of MAPP, which maintains both the stiffness and toughness of the material.

#### 3.1.2. Micro-Morphological Analysis of MAPP-Modified GFRP/WPC

**Figure 2:**
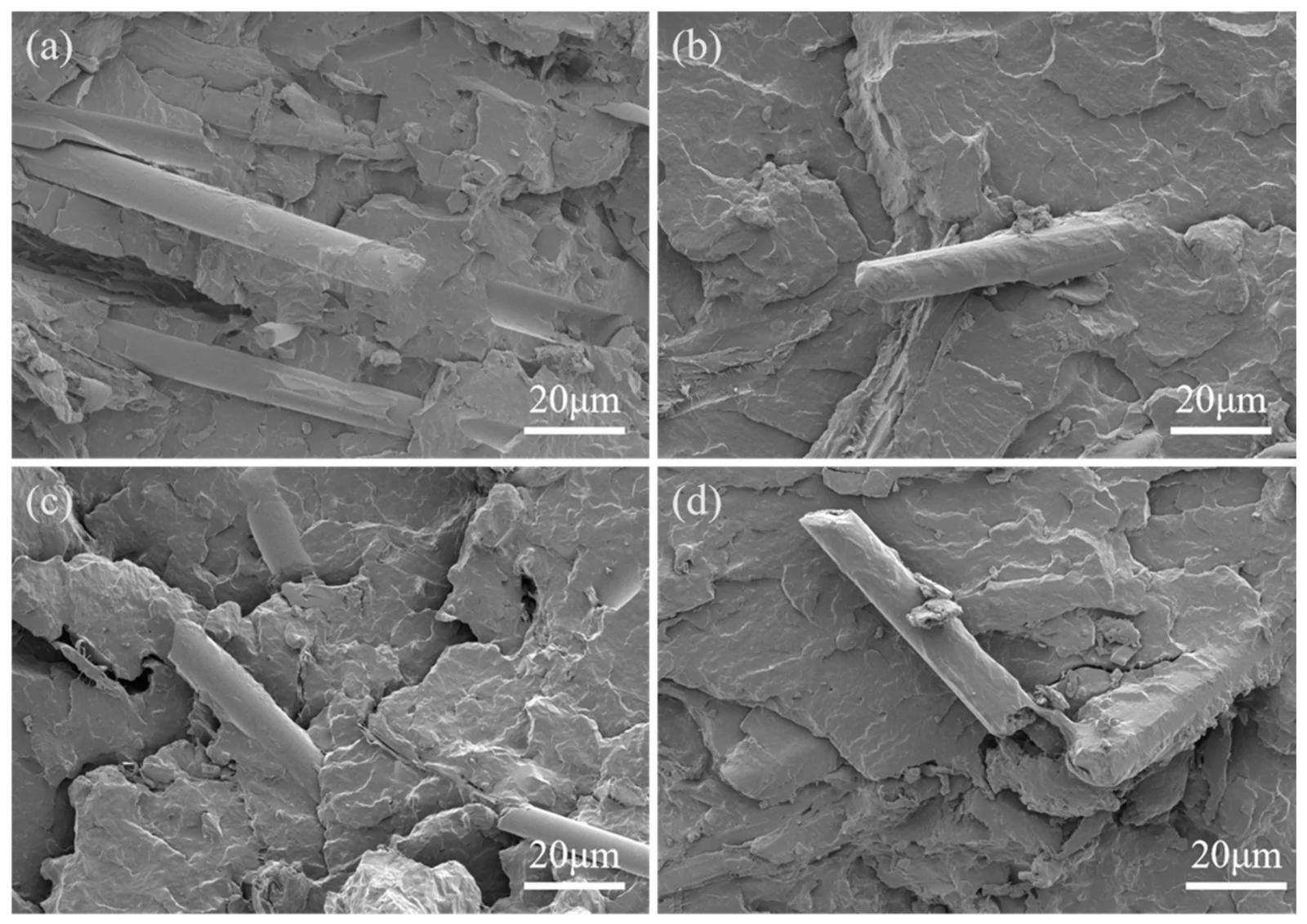
SEM images of the cross-sectional morphology of MAPP-modified GFRP/WPC: (**a**) control; (**b**) MAPP-1; (**c**) MAPP-3; (**d**) MAPP-7.

Figure 2 shows SEM images of the fracture surfaces of MAPP-modified specimens at 500× magnification. Figure 2a corresponds to the unmodified sample, where many holes left by pulled-out GFRP fibers can be observed. In addition, the bonding between GFRP and the matrix resin is weak, with visible gaps at the interface. Under stress, energy is dissipated by fiber pull-out from the plastic matrix, which is one reason why the improvement in mechanical properties of GFRP/WPC by GFRP powder is limited. From Figure 2b,c, it can be seen that after MAPP addition, the fracture surface of GFRP/WPC becomes smoother and the bonding with the matrix resin is stronger. The failure mode changes from fiber pull-out to fiber fracture, indicating enhanced interfacial adhesion of the GFRP/WPC material.

However, with further increase in MAPP, as seen in Figure 2d, although the fibers in GFRP are well coated, excessive MAPP remains free between the matrix resin and fibers, leading to a decline in mechanical properties. Therefore, it can be confirmed that 3% MAPP gives the best performance. These observations are consistent with the mechanical test results.

#### 3.1.3. Surface Free Energy of MAPP-Modified GFRP/WPC

The change in surface free energy of GFRP/WPC can reflect its interfacial adhesion. From Table 5, it can be seen that after MAPP modification, the water contact angle and ethylene glycol contact angle decrease slightly but not significantly. The surface free energy of GFRP/WPC increases slightly, with the most obvious increase in the polar component. Taking MAPP-3 as an example, the polar component increases by 36.13% compared with the unmodified GFRP/WPCs.

The significant increase in the polar component is due to the fact that the matrix resin surface is mainly composed of methyl groups with low polarity, and the surface free energy is primarily contributed by the dispersive component. After MAPP modification, MAPP acts as a “bridge” to effectively connect the reinforcing fibers with the matrix resin, resulting in better encapsulation. This causes the surface of the modified GFRP/WPC to be more dominated by the matrix resin and MAPP. Since MAPP itself is rich in polar functional groups, these groups exposed on the surface greatly increase the interaction between the GFRP/WPC surface and the test liquids, leading to a sharp rise in the measured polar component [26].

#### 3.1.4. Thermal Properties of MAPP-Modified GFRP/WPC

**Figure 3:**
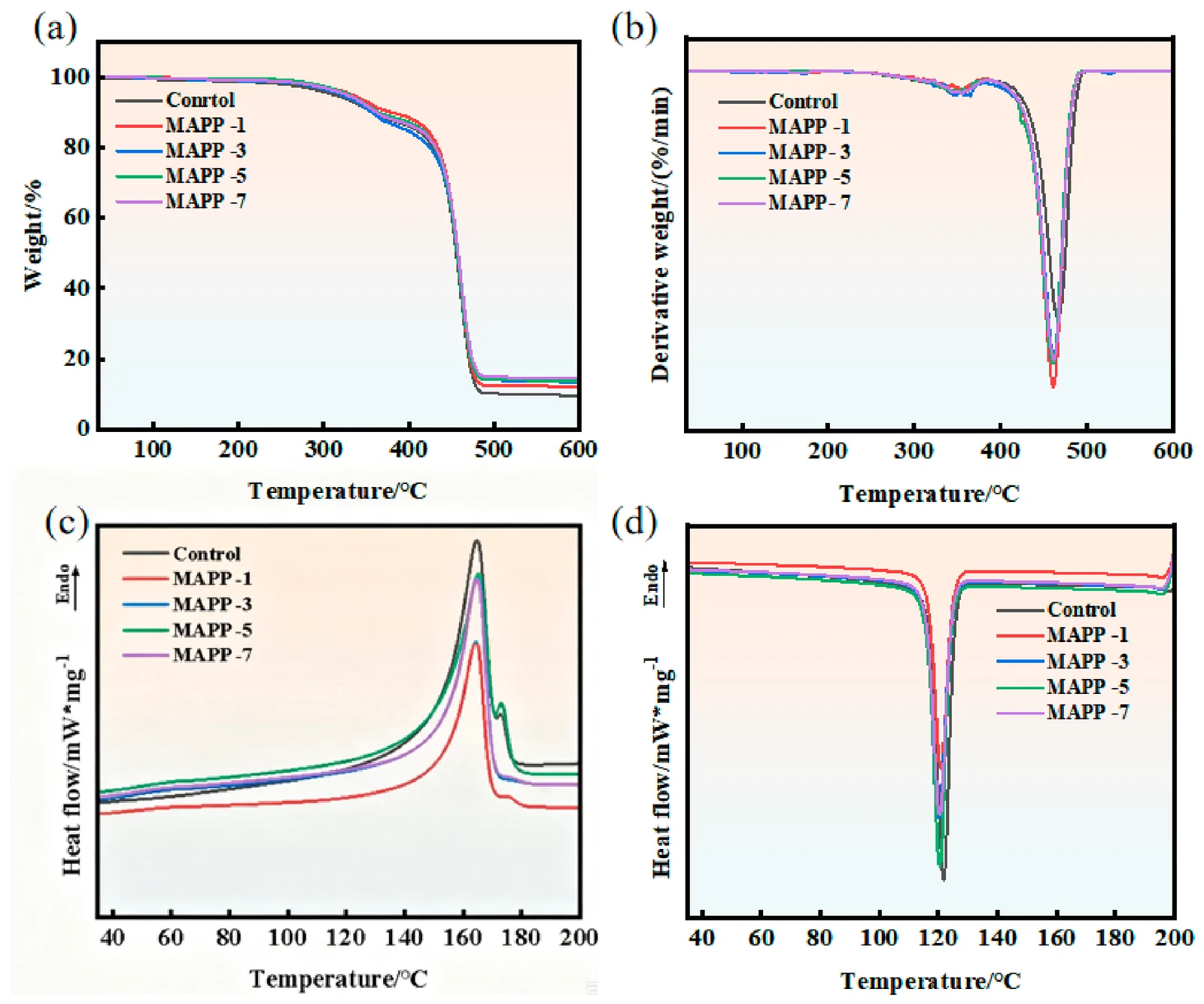
MAPP-modified GFRP/WPCs: (**a**) TG curves; (**b**) DTG curves; (**c**) melting diagram; (**d**) crystallization behavior.

Figure 3a,b show the TG curves of MAPP-modified GFRP/WPCs, and Table 6 lists the key parameters during thermal decomposition. The pyrolysis process of GFRP/WPCs still exhibits three stages, indicating that MAPP has little effect on the overall pyrolysis behavior. In terms of thermal decomposition parameters, it can be seen that after MAPP treatment, $T_{5wt\%}$ and $T_{max}$ are slightly lower than those of the unmodified sample. This is because after MAPP modification, the matrix resin tightly encapsulates the GFRP powder and wood flour; during heating, the surface matrix resin decomposes first. Similarly, after decomposition, the matrix resin protects the glass fiber fraction, reducing porosity and leading to incomplete pyrolysis, so that most of the glass fiber components remain, resulting in increased residue at 600 °C.

Figure 3c,d show the heating and cooling curves of MAPP-modified GFRP/WPCs from DSC measurements. After MAPP modification, the melting temperature ($T_{m}$) increases slightly, while the crystallization temperature ($T_{c}$) decreases slightly. This is because MAPP restricts the flow of macromolecular chains in the matrix resin, so that movement occurs only at higher temperatures. In addition, the crystallinity of GFRP/WPCs increases from 50.85% up to 59.81%, which can be attributed to the fact that MAPP modification enhances the mobility of polymer molecular chains, promotes the formation of nucleation sites with GFRP and wood flour, and accelerates the crystallization rate, thus leading to an overall increase in crystallinity.

It is worth noting that the crystallinity does not exhibit a strictly monotonic trend with increasing MAPP content (Table 6). The crystallinity initially decreases at 1 wt% MAPP (47.39%) compared to the control (50.85%), then increases to its maximum at 5 wt% MAPP (59.81%) before decreasing again at 7 wt% MAPP (55.24%). This non-monotonic behavior can be attributed to the competing effects of MAPP on PP crystallization. At low MAPP content (1 wt%), the compatibilizer may not be sufficient to form effective nucleation sites, while it may also slightly plasticize the matrix, reducing crystallinity. As MAPP content increases to 3–5 wt%, the nucleating effect dominates, promoting crystal formation and increasing crystallinity. However, at excessive MAPP content (7 wt%), the excess compatibilizer may act as a diluent or disrupt chain mobility, leading to a reduction in crystallinity. This competitive mechanism has been observed in other compatibilized polymer blend systems.

### 3.2. Effect of H_2_O_2_ Treatment on Properties of GFRP/WPC

#### 3.2.1. Characterization of H_2_O_2_-Treated GFRP Powder

**Figure 4:**
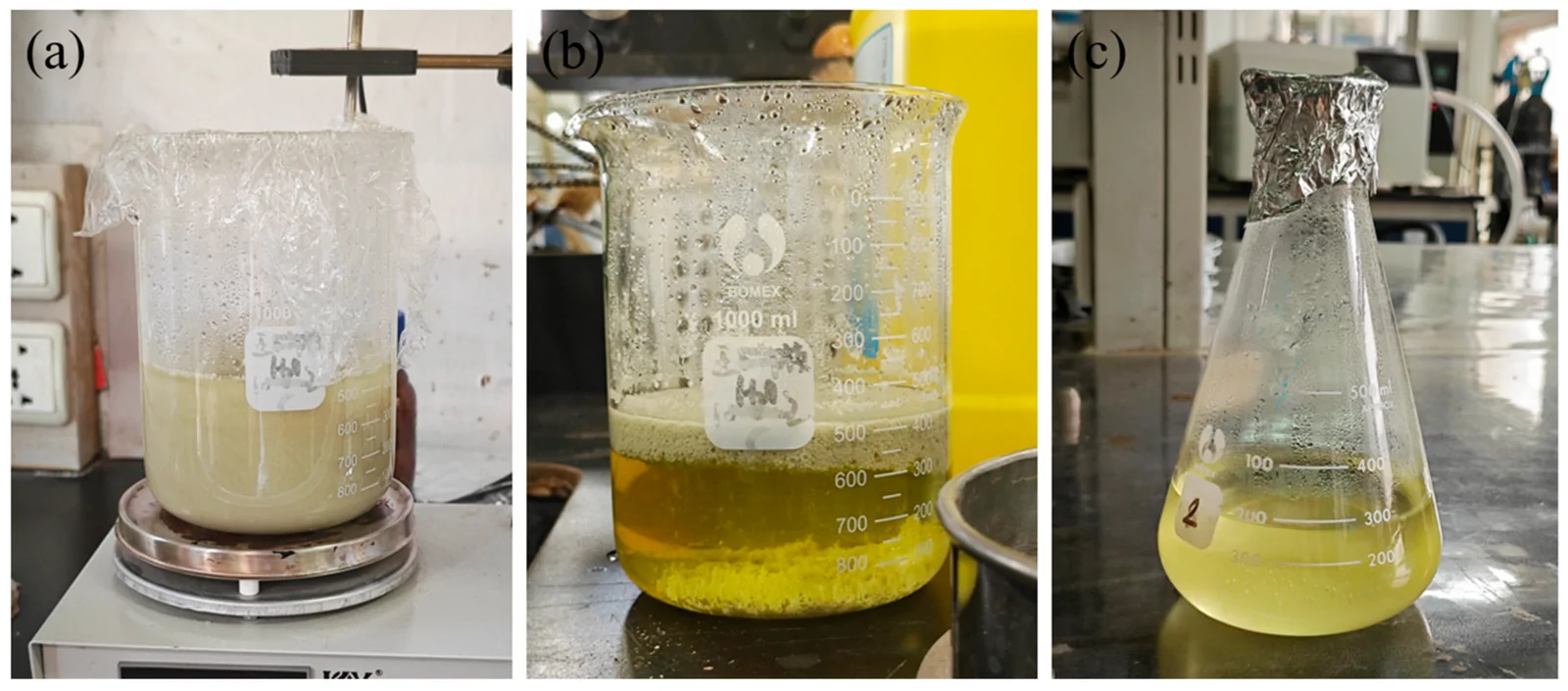
Photographs of the H_2_O_2_ treatment process for GFRP powder: (**a**) GFRP immersed in H_2_O_2_; (**b**) the solution after 6 h of treatment; (**c**) the liquid product after decomposition.

As shown in Figure 4a, GFRP powder is thoroughly mixed in H_2_O_2_ under stirring. As the reaction time increases, the solution turns yellow and powdered GFRP floats on top, while the color of GFRP does not change significantly due to oxidation. In Figure 4b, after 6 h of reaction and standing, the GFRP powder separates from the treatment solution, indicating that decomposition products are formed after H_2_O_2_ treatment.

**Figure 5:**
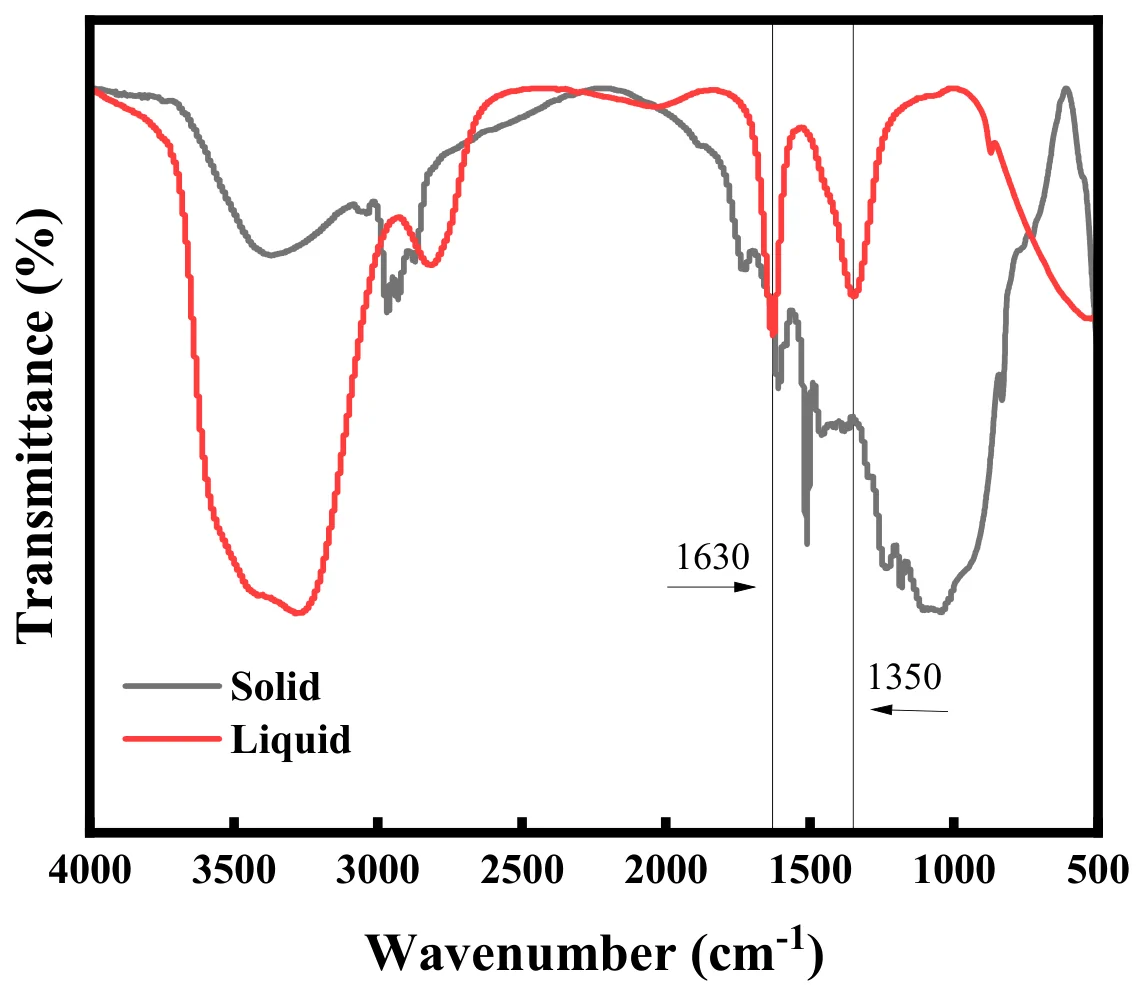
Infrared spectra of the liquid and solid components in the decomposition products.

Figure 5 shows the FTIR spectra of the decomposition products. For the liquid product, peaks at 1347.53 cm^−1^ and 1628.75 cm^−1^ indicate the presence of phenolic ketones and carbonyl ketones in the recovered liquid. The broad peak around 3285.47 cm^−1^ is likely due to interference from water molecules. During the oxidation process with hydrogen peroxide, the epoxy resin in the GFRP powder is mainly degraded into a mixture of bisphenol A derivatives, phenolic derivatives, and ketone or carboxyl-containing molecules [18]. The presence of these functional groups indicates that hydrogen peroxide can partially decompose the epoxy resin in GFRP, and the products show some potential for recycling.

**Figure 6:**
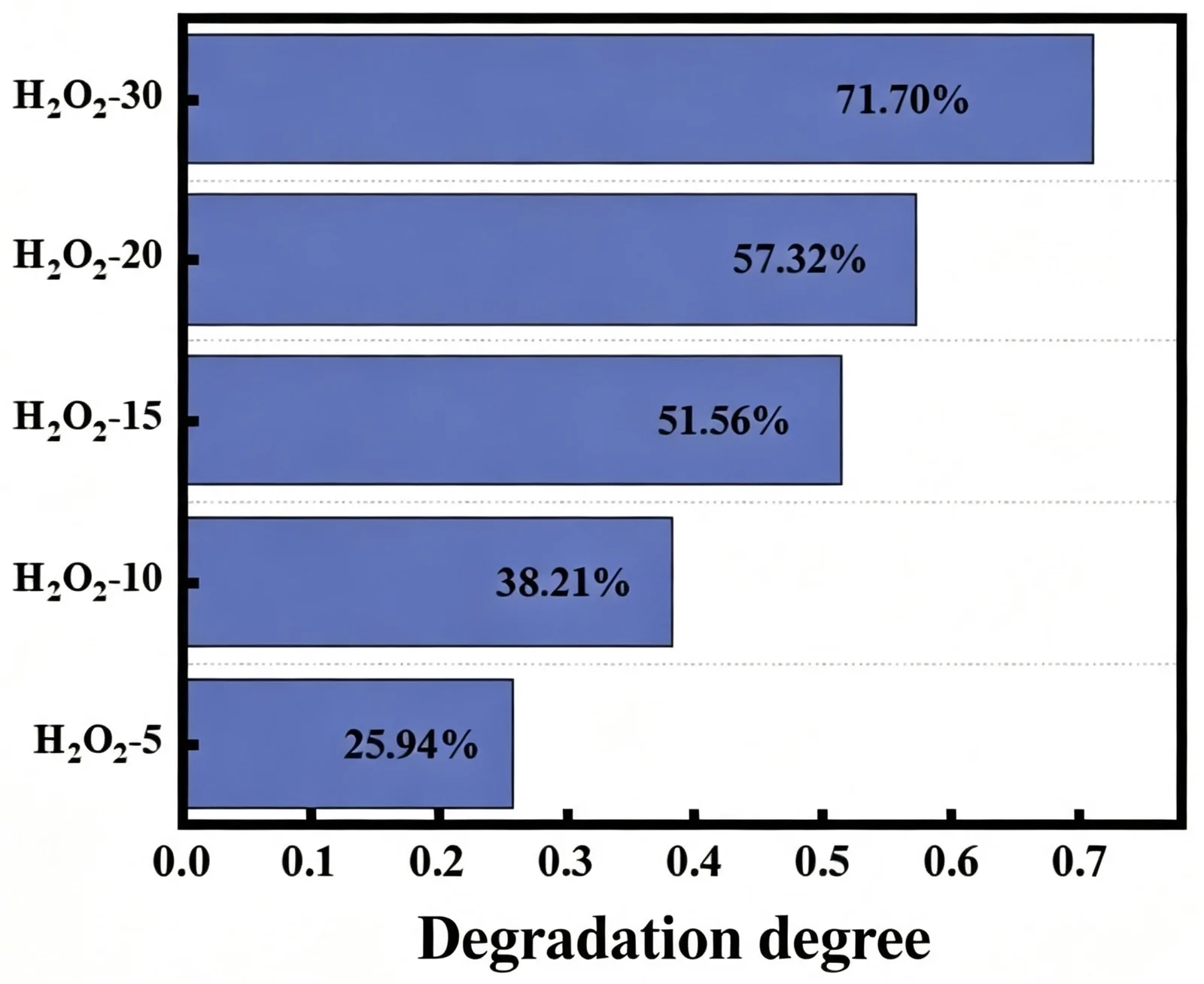
Degradation degree of GFRP powder by different H_2_O_2_.

As shown in Figure 6, the mass of GFRP powder decreases after H_2_O_2_ treatment, and the degradation degree increases with increasing H_2_O_2_ concentration, reaching 71.70% at 30% concentration. This value represents the mass loss of the organic epoxy resin component relative to the initial epoxy content (23.03 wt% of the pristine powder). The observed degradation behavior is consistent with the FTIR results (Figure 5), which confirmed the progressive oxidative breakdown of the epoxy resin into soluble fragments. The slight deviation from complete removal (100%) at 30% H_2_O_2_ suggests that a fraction of the epoxy resin may be tightly bound to the glass fiber surface or embedded within the fiber bundles, making it less accessible to oxidative degradation. This residual epoxy, though reduced, may still contribute to some interfacial bonding in the final composite. Combined with the FTIR analysis, the mass loss is mainly due to the decomposition of epoxy resin, accompanied by a small loss of glass fibers.

#### 3.2.2. Effect of H_2_O_2_ Treatment on Mechanical Properties of GFRP/WPC

**Figure 7:**
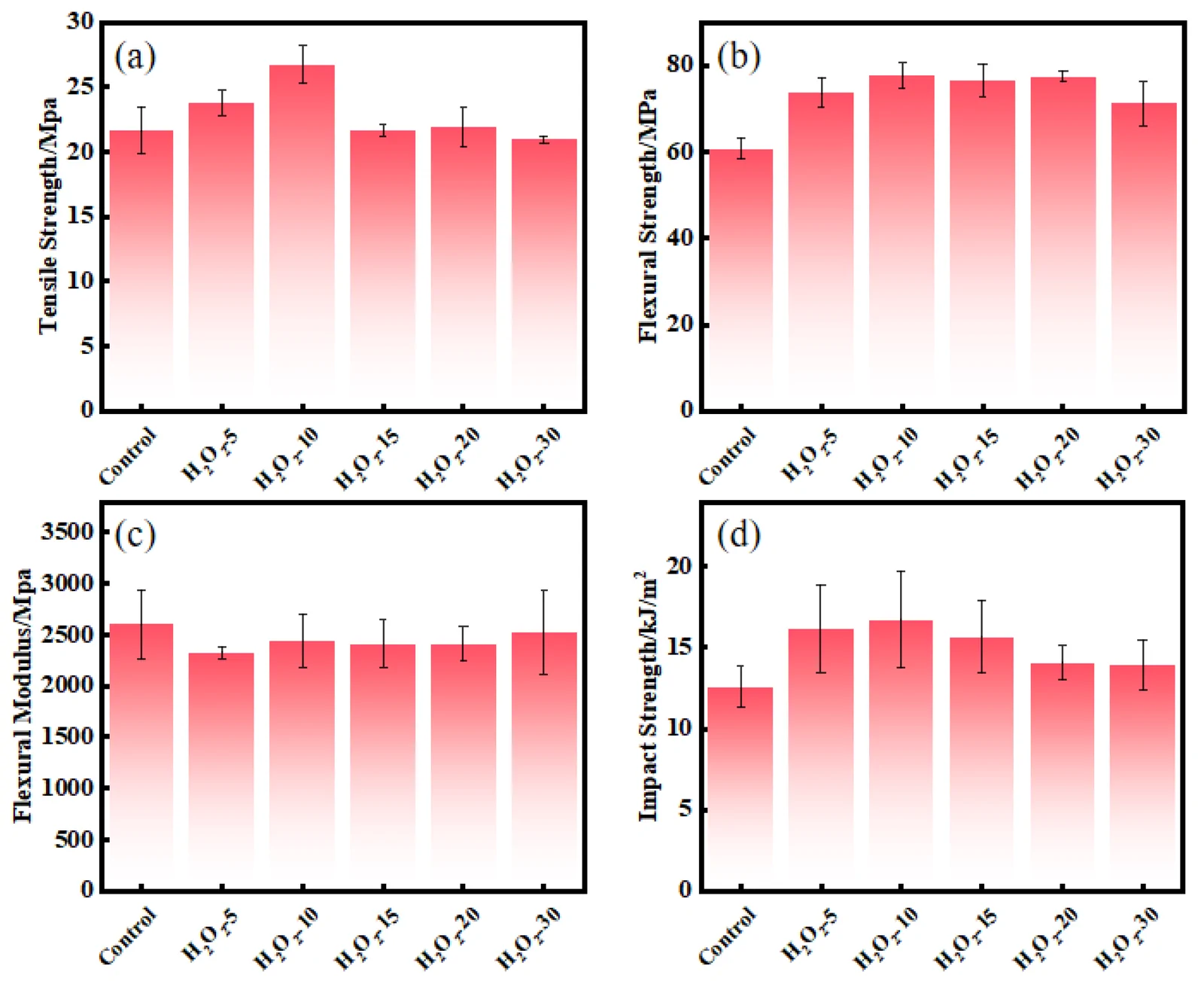
Mechanical Properties of H2O2-Modified GFRP/WPC: (**a**) tensile strength; (**b**) flexural strength; (**c**) flexural modulus; (**d**) impact strength.

Figure 7 shows the mechanical properties of GFRP/WPC after modification with different concentrations of H_2_O_2_. It can be seen that the tensile strength, flexural strength and impact strength of GFRP/WPC are improved, while the flexural modulus decreases. The tensile strength, flexural strength and impact strength reach their maxima at a H_2_O_2_ concentration of 10%, with values of 26.7 MPa, 77.4 MPa and 17.0 kJ/m^2^, respectively, which are 23.65%, 27.93% and 35.29% higher than those of the unmodified GFRP/WPC (21.57 MPa, 60.53 MPa and 12.55 kJ/m^2^, respectively). The flexural modulus reaches its minimum of 2322.5 MPa at 5% H_2_O_2_, representing a 10.5% decrease compared with the unmodified sample. After H_2_O_2_ modification, all mechanical properties are improved, and except for flexural modulus, the mechanical properties of H_2_O_2_-modified GFRP/WPCs are superior to those of MAPP-modified GFRP/WPCs.

However, at H_2_O_2_ concentrations above 10%, the impact strength began to decline, which can be attributed to the embrittlement caused by excessive oxidation of the glass fibers. The removal of the protective epoxy layer at high treatment levels may expose glass fibers to surface defects, reducing their ability to absorb impact energy. This suggests that while moderate H_2_O_2_ treatment enhances toughness, excessive treatment leads to a brittle interface and reduced impact performance.

In terms of mechanical properties, after H_2_O_2_ treatment, the surface and free epoxy resin components on GFRP are destroyed, eliminating the weak interfacial layer between the epoxy resin and the matrix resin, while the main reinforcing phase—glass fibers—is retained. In the composite, glass fibers can act as a “skeleton”. When the material bears stress, the glass fibers effectively transfer stress and maintain the strength of the material. When the epoxy resin component is removed from the GFRP powder, the weak interfacial layer between the glass fibers and the matrix resin is reduced, the contact area with the matrix resin increases, and an interlocking structure is formed with the wood fibers. Thus, stress can be more effectively transferred from the plastic matrix to the high-strength glass fibers, enabling the fibers to bear the load and dissipate energy through load-bearing rather than pull-out, thereby improving the strength and toughness of GFRP/WPC.

At the same time, after removing part of the epoxy resin, the flexural modulus decreases. The reduction in flexural modulus is related to the removal of the epoxy resin component from the GFRP powder. The thermosetting resin, due to its rigid shape, acts as a filler that generates stress to resist bending deformation, increasing the overall stiffness and reducing the elasticity of GFRP/WPC.

The decline in mechanical properties above 10 wt% H_2_O_2_ and the reductions in surface energy and thermal stability at higher concentrations suggest possible oxidative damage to the glass fibers, which has been reported to occur under strongly oxidizing conditions [7,8]. However, we acknowledge that direct observations—such as SEM imaging of fiber surfaces, single fiber tensile strength testing, or fiber diameter/length distribution analysis—were not performed in the current study due to practical constraints. Therefore, the proposed mechanism, while plausible based on indirect evidence and prior literature, should be confirmed in future investigations.

One-way ANOVA followed by Tukey’s HSD post hoc test was performed to validate the statistical significance of the mechanical properties. The results confirmed that the improvements at 10% H_2_O_2_ for tensile strength, flexural strength, and impact strength were statistically significant (*p* < 0.05) compared to the control. The reduction in flexural modulus at 5% H_2_O_2_ was also statistically significant (*p* < 0.05), confirming the trade-off between strength enhancement and stiffness loss.

In summary, H_2_O_2_ treatment has a positive effect on GFRP powder by removing part of the epoxy resin component, and the greatest improvement in mechanical properties is achieved at a concentration of 10%.

#### 3.2.3. Micro-Morphology of H_2_O_2_-Treated GFRP/WPC

**Figure 8:**
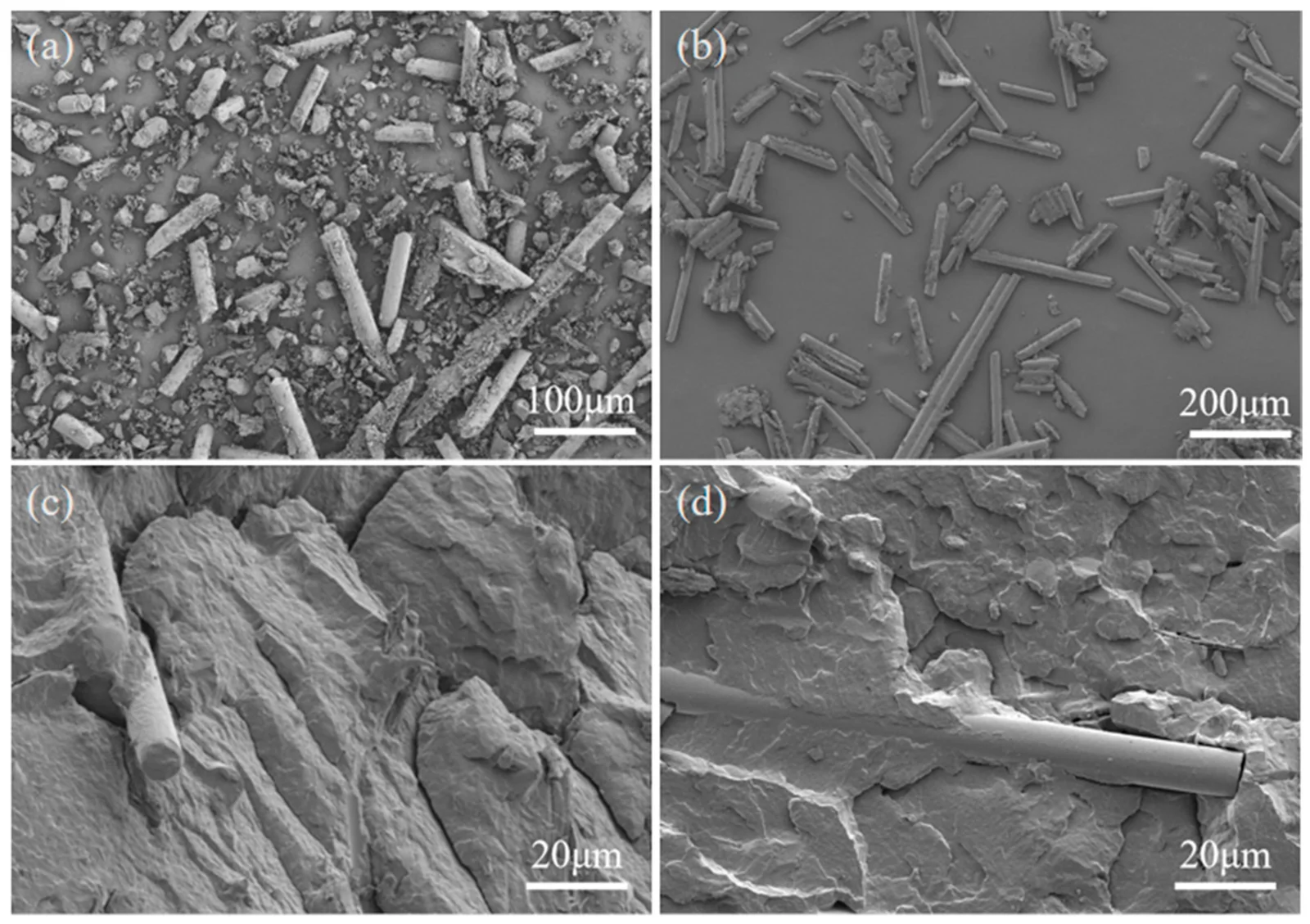
(**a**) GFRP powder before modification; (**b**) GFRP powder after modification; (**c**,**d**) SEM image of cross-sectional morphology of H_2_O_2_-modified GFRP/WPC: (**c**) H_2_O_2_-10; (**d**) H_2_O_2_-15.

Comparing Figure 8a,b, it can be seen that unlike the untreated GFRP powder, the H_2_O_2_-modified powder surface is smoother, and irregular epoxy resin fragments are significantly reduced, although some still remain. Figure 8c and Figure 8d show the fracture surfaces of GFRP/WPC modified with 10% and 15% H_2_O_2_, respectively. Unlike the gaps between glass fibers and matrix resin seen in Figure 2a, the H_2_O_2_-modified GFRP/WPC clearly shows that the glass fibers are almost embedded in the matrix, with good interfacial bonding. There is no obvious fiber pull-out on the fracture surface. This allows the glass fiber component in GFRP to better play its reinforcing role under load, enhancing the interfacial bonding and further bearing the load. Macroscopically, this is reflected in a significant improvement in the strength and toughness of GFRP/WPC. At the same time, the free epoxy resin at the interface is reduced, and the original rigid filler effect in the GFRP/WPC system is weakened; so, the resistance to small deformation decreases, manifesting as a reduction in flexural modulus.

From the SEM images presented in Figure 8, visual inspection of the H_2_O_2_-treated GFRP powder reveals that the glass fiber length ranges approximately from 20 to 80 μm, while the fiber diameters are roughly 5–15 μm. These values are consistent with the typical fiber dimensions reported for mechanically recycled wind turbine blade waste [7,8]. Notably, no apparent fiber shortening or severe surface damage was observed at the optimal H_2_O_2_ concentration (10%), suggesting that the oxidative treatment does not significantly compromise the structural integrity of the glass fibers. However, systematic quantitative analysis of fiber length and diameter distributions will be performed in future work to provide more precise statistical validation.

#### 3.2.4. Surface Free Energy of H_2_O_2_-Treated GFRP/WPC

The contact angle and surface free energy of GFRP/WPC after H_2_O_2_ treatment are shown in Table 7. It can be observed that after H_2_O_2_ treatment, the distilled water contact angle on the material surface increases significantly, by 11.48% compared with the untreated group. The ethylene glycol contact angle increases slightly but not substantially. The surface free energy of GFRP/WPC all increases markedly, with the polar component first increasing and reaching a maximum of 0.869 at 10% H_2_O_2_. The dispersive component also reaches its maximum at 10% H_2_O_2_. It is important to note that the surface free energy measurements were conducted on compression-molded composite sheets, where the exposed surface is predominantly the PP matrix. Nevertheless, the measured surface energy of such composite surfaces reflects the combined effects of the matrix and the underlying filler chemistry, as previously established in the literature [27,28,29].

The changes in surface free energy observed on the composite surface reflect the altered surface chemistry of the embedded GFRP powder following H_2_O_2_ treatment. Although the exposed surface of compression-molded specimens is dominated by the PP matrix, the surface free energy measured on such composite surfaces is known to be influenced by the underlying filler surface chemistry through changes in the matrix-filler interphase and the resulting surface morphology [27,28]. The removal of the polar epoxy layer from the GFRP surface reduces the overall polarity of the filler, which in turn affects the wetting behavior and surface energy of the composite. This interpretation is consistent with previous studies on filled polymer composites, where modifications to the filler surface were shown to alter the composite surface free energy even when the filler was not directly exposed on the surface [29]. Furthermore, the observed increase in the dispersive component after H_2_O_2_ treatment can be attributed to the exposure of the inherently hydrophobic glass fiber surfaces (rich in Si–O–Si structures) within the composite, which reduces the polar contribution to the overall surface energy. It should be noted that direct measurement of the treated GFRP powder by powder contact angle or inverse gas chromatography would provide additional verification; however, such measurements were beyond the scope of the present study and are suggested for future investigation. However, when the H_2_O_2_ concentration is too high, the contact angle and polar component decrease; excessively damaged glass fibers have reduced strength, and the molten matrix resin cannot wet their surface well, causing defects on the GFRP/WPC surface and a decrease in surface free energy, which in turn leads to a decline in overall mechanical properties, consistent with the mechanical property trends.

#### 3.2.5. Thermal Properties of H_2_O_2_-Treated GFRP/WPC

**Figure 9:**
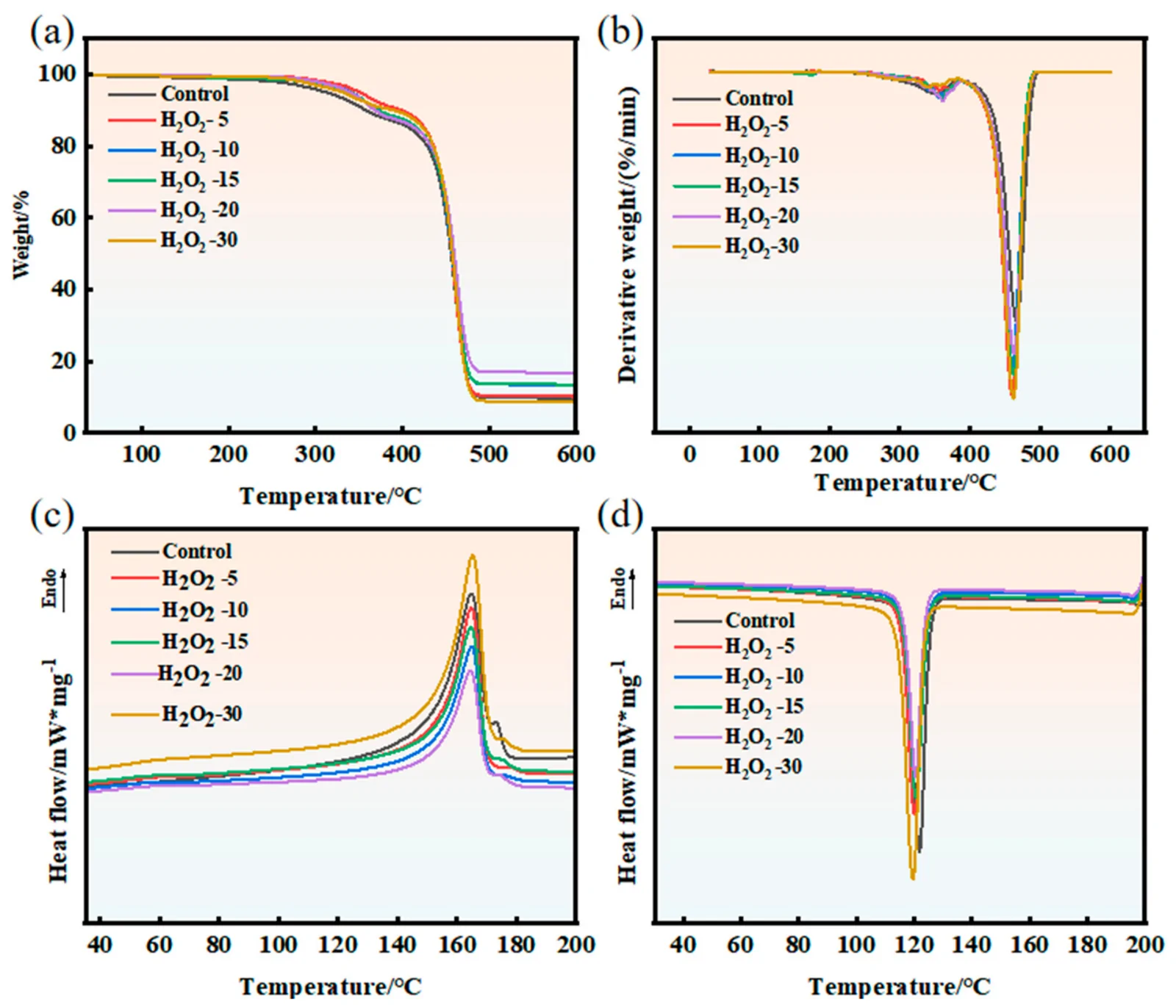
H_2_O_2_-modified GFRP/WPC: (**a**) TG curves; (**b**) DTG curves; (**c**) melting diagram; (**d**) crystallization behavior.

Figure 9a,b show the TG curves of H_2_O_2_-modified GFRP/WPC, and Table 8 lists the key thermal decomposition parameters. It can be seen that H_2_O_2_ has little effect on the basic thermal decomposition behavior of GFRP/WPCs, which still proceeds in three stages. $T_{5wt\%}$ increases while $T_{max}$ decreases slightly. This is because after H_2_O_2_ modification, the proportion of glass fiber components in GFRP is higher, improving heat resistance and leading to a higher initial decomposition temperature. However, when the H_2_O_2_ concentration exceeds 20%, the thermal decomposition temperature decreases, possibly due to excessive damage to the glass fiber surface by H_2_O_2_, creating micropores in the matrix resin that accelerate heat transfer in GFRP/WPCs.

Figure 9c,d show the heating and cooling curves of H_2_O_2_-modified GFRP/WPC from DSC. After H_2_O_2_ modification, the melting temperature ($T_{m}$) increases from 164.7 °C to 165.1 °C, while the crystallization temperature decreases slightly, and the crystallinity of GFRP/WPC increases. After H_2_O_2_ modification, the epoxy resin and other low-molecular-weight compounds on the glass fiber surface are removed, exposing a smoother glass fiber surface that can more effectively form nucleation sites with the matrix resin, leading to more regular chain entanglement and arrangement.

Similarly, for H_2_O_2_-modified GFRP/WPCs, the crystallinity does not follow a strict linear trend with increasing H_2_O_2_ concentration (Table 8). The crystallinity increases from the control (50.85%) to H_2_O_2_-15 (55.82%), then decreases at H_2_O_2_-20 (50.02%) before rising again at H_2_O_2_-30 (55.06%). This variation may reflect the competing effects of two mechanisms: (1) the removal of epoxy resin from the glass fiber surface exposes cleaner nucleation sites, promoting PP crystallization; and (2) excessive H_2_O_2_ treatment may damage glass fibers or create surface defects, reducing their nucleating efficiency. The interplay of these two factors results in the observed non-monotonic crystallinity trend.

Melt flow index and viscosity measurements were not performed in this study, as the primary focus was on solid-state mechanical properties and interfacial chemistry. However, it is acknowledged that interfacial modifications inevitably affect melt viscosity, which is critical for real-world extrusion and injection molding processes. Future work should include rheological characterization to optimize processing conditions and ensure industrial applicability.

The sustainability of the H_2_O_2_ treatment route requires consideration of its liquid by-products. As shown in Figure 5, the decomposition liquid contains phenolic and carbonyl species, including bisphenol A derivatives, which are toxic and require proper treatment before disposal. Possible strategies include neutralization, activated carbon adsorption, or advance2d oxidation processes [30,31]. Additionally, the H_2_O_2_ process requires heating at 90 °C for 6 h followed by washing and drying (along with neutralization steps), consuming substantially more energy than the direct melt-blending MAPP approach. Thus, while H_2_O_2_ treatment provides superior strength and toughness, its environmental benefits must be balanced against higher energy consumption and waste treatment burdens. A full life cycle assessment (LCA) or detailed cost analysis was beyond the scope of the present study. However, based on the preliminary energy comparison and the simplicity of the MAPP process, it is evident that MAPP offers a more sustainable and economically viable alternative for applications not requiring maximum strength. Future work should include a comprehensive LCA to quantitatively compare the environmental footprints of the two modification routes.

While the present study focuses on the mechanical and interfacial properties of freshly prepared composites, the moisture durability of GFRP/WPCs is certainly important for outdoor construction applications. Although water absorption and thickness swelling tests were not performed in this study due to limited access to long-term testing facilities, previous studies on similar WPC systems incorporating recycled glass fibers have reported that the hydrophobic nature of the PP matrix and the glass fiber components inherently limits water uptake [5,6,12]. Additionally, the remaining epoxy resin on the GFRP surface, even after H_2_O_2_ treatment, may still provide a degree of hydrophobic protection. Given the encouraging mechanical performance observed in the present work, future studies will systematically evaluate the long-term water resistance and dimensional stability of these optimized formulations.

It should be acknowledged that the rGFRP powder used in this study was obtained from a single source (decommissioned wind turbine blades from a specific batch). The properties of rGFRP waste—such as fiber length distribution, residual epoxy content, and glass fiber surface condition—can vary depending on the original blade manufacturer, service history, and recycling process. Such variability may affect the reproducibility of the results. While the mechanical recycling process employed in this work provides a consistent powder fraction (S-GFRP, <100 mesh), future studies should investigate the performance of the optimized formulations using rGFRP from different sources to establish the robustness and general applicability of the findings.

The findings of this study are consistent with recent international research on the valorization of recycled wind turbine blade waste. For instance, Zhang et al. [12] demonstrated that recycled GFRP from decommissioned wind turbine blades could be effectively incorporated into wood-based composites, achieving enhanced mechanical properties through gradient structural design. Similarly, Hao et al. [9] reported that mild chemical recycling of wind turbine blade waste enabled direct reuse in thermoplastic composites with improved performance. The use of MAPP as a compatibilizer in recycled fiber-reinforced composites has also been extensively studied; for example, Poletto et al. [15] and Fatima Ezzahrae et al. [16] confirmed its effectiveness in improving interfacial adhesion in wood-polymer systems. Furthermore, the application of oxidative treatments such as H_2_O_2_ to modify recycled fiber surfaces has been explored by Petropoulos et al. [18], who demonstrated epoxy removal from glass fiber surfaces, consistent with our FTIR observations. Collectively, these studies support our findings and highlight the potential of rGFRP as a sustainable reinforcement for WPCs.

It should be noted that WPCs and rGFRP are known to be sensitive to moisture and UV radiation in outdoor service environments. While the present study focuses on the mechanical and interfacial properties of freshly prepared composites, the lack of accelerated weathering or hygrothermal aging data is a limitation. Long-term durability is certainly important for practical applications, and future work will include systematic evaluation of weathering resistance and moisture aging performance to ensure the suitability of these composites for outdoor construction use. This is a clear direction for our ongoing research.

## 4. Conclusions

In this study, two modification strategies were applied to enhance the performance of recycled glass fiber-reinforced plastic (GFRP)/wood flour/polypropylene (PP) composites: (1) the addition of maleic anhydride grafted polypropylene (MAPP) as a compatibilizer, and (2) hydrogen peroxide (H_2_O_2_) treatment of GFRP powder to remove the epoxy resin coating. All composites were prepared with fixed contents of 10 wt% wood flour and 15 wt% GFRP via internal mixing and hot-pressing. The effects of these modifications on the mechanical properties, micro-morphology, surface free energy, thermal stability and crystallization behavior were systematically investigated. The main conclusions are summarized as follows:

1. Both modification methods effectively improved the overall mechanical properties of the composites, with optimal values obtained at 3 wt% MAPP and 10 wt% H_2_O_2_, respectively. MAPP modification improved the mechanical properties, though the optimal concentration differed by property. Flexural strength was maximized at 1 wt% MAPP (75.45 MPa, +24.7%), while tensile strength (+9.2%), flexural modulus (+20.7%), and impact strength (+18.9%) reached their maxima at 3 wt% MAPP. At 10 wt% H_2_O_2_, the tensile strength, flexural strength and impact strength were enhanced by 23.65% (to 26.7 MPa), 27.93% (to 77.4 MPa) and 35.29% (to 16.98 kJ/m^2^), respectively; although the flexural modulus decreased slightly (by approximately 10.5% at 5% H_2_O_2_), the tensile and impact strengths were significantly enhanced. This trade-off—strength improvement at the cost of some stiffness—should be considered when selecting the appropriate modification strategy for specific applications.
2. H_2_O_2_ treatment effectively decomposed and removed the epoxy resin from the GFRP surface, as confirmed by FTIR analysis of the waste liquid, with a maximum removal rate of 71.7%. The removal of this weak interfacial layer improved the wetting and adhesion between the exposed glass fibers and the PP matrix.
3. Both modifications had only a minor influence on the thermal decomposition behavior of the composites. The initial decomposition temperature decreased slightly, but the residual mass at 600 °C increased owing to better encapsulation of the glass fibers. Regarding crystallization, MAPP enhanced the crystallinity by promoting molecular chain mobility and providing nucleation sites through encapsulation of the reinforcements, whereas H_2_O_2_ treatment produced a smoother glass fiber surface, which increased the number of nucleation sites and thus raised the overall crystallinity.
4. SEM observations of the fracture surfaces revealed that MAPP modification led to tighter interfacial bonding among GFRP, wood flour and the matrix resin. In contrast, H_2_O_2_ treatment significantly reduced the epoxy resin attached to the GFRP surface and the free epoxy resin, resulting in improved interfacial adhesion between the glass fiber component and the PP matrix.
5. Surface free energy measurements showed that both modification methods increased the surface free energy and the polar component of the composites. MAPP modification caused little change in contact angle, with the increase in surface free energy mainly attributable to the polar component. In the case of H_2_O_2_ modification, the contact angle increased noticeably, and both polar and dispersive components rose significantly. However, excessive H_2_O_2_ concentration caused over-damage to the glass fibers, leading to reduced wettability and a consequent decline in mechanical properties.

In summary, both MAPP compatibilization and moderate H_2_O_2_ treatment are effective approaches for improving the interfacial adhesion and overall performance of GFRP/WPCs. The findings provide useful guidance for the valorization of recycled GFRP in wood–plastic composite applications.

## Figures and Tables

**Table 1:** Experimental materials.

Raw Material	Specification/Model	Manufacturer
Poplar wood flour (WF)	100 mesh	Linyi Tianlai Zhilin Wood Industry Co., Ltd. (Linyi, China)
Recycled GFRP powder	S-GFRP	Fengnuo (Jiangsu) Environmental Protection Technology Co., Ltd. (Yancheng, China)
Polypropylene resin (PP)	Grade: S1003; MFI: 3.14 g/10 min	Zhejiang Petroleum & Chemical Co., Ltd. (Zhoushan, China)
Maleic anhydride grafted polypropylene (MAPP)	Grafting level: 1.0 wt%; MFI: 30 g/10 min	Dongguan Dinghai Plastic Chemical Co., Ltd. (Dongguan, China)
Hydrogen peroxide	30%	Shanghai Aladdin Biochemical Technology Co., Ltd. (Shanghai, China)
Deionized water	Grade I	Laboratory-prepared

**Table 2:** Experimental equipment.

Equipment	Model	Manufacturer
Electronic balance	AUX220	Shimadzu Corporation, Kyoto, Japan
Pressurized internal mixer	ZG-0.5L	Dongguan Zhenggong Electromechanical Equipment Technology Co., Ltd., Dongguan, China
100 T universal press	BY302X2/2	Suzhou Xinxieli Co., Ltd., Suzhou, China
Cold press	CGYJ-100	Shijiazhuang Cangao High-frequency Machinery Co., Ltd., Shijiazhuang, China
Electric forced-air drying oven	TH-881Y-5	Suzhou Yihua Oven Equipment Co., Ltd., Suzhou, China
Magnetic heating stirrer	HJ-4	Jintan Kexi Instrument Co., Ltd., Jintan, China
Microcomputer-controlled universal wood testing machine	MWW-100E	Jinan Nai’er Testing Machine Co., Ltd., Jinan, China
Electronic cantilever beam impact tester	XJJD-5	Beijing Building Materials Inspection and Research Institute Co., Ltd., Beijing, China
Fourier-transform infrared spectrometer (FTIR)	Nicolet iS20	Thermo Scientific, Waltham, MA, USA
High-resolution field-emission scanning electron microscope (SEM)	Regulus8100	Hitachi, Tokyo, Japan
Thermogravimetric analyzer (TG)	HITACHI STA200	Hitachi, Tokyo, Japan
Differential scanning calorimeter (DSC)	DSC200	Hitachi, Tokyo, Japan
Contact angle goniometer	SL200KS	KINO Industry Co., Somerville, MA, USA

**Table 3:** Formulation for MAPP-modified GFRP/WPC.

Sample ID	MAPP (wt%)	PP (wt%)
Control	0	75
MAPP-1	1	74
MAPP-3	3	72
MAPP-5	5	70
MAPP-7	7	68

**Table 4:** Formulation for H_2_O_2_-modified GFRP/WPC.

Sample ID	H_2_O_2_ (wt%)	PP (wt%)	Wood Flour (wt%)	S-GFRP (wt%)
Control	–	75	10	15
H_2_O_2_-5	5	75	10	15
H_2_O_2_-10	10	75	10	15
H_2_O_2_-15	15	75	10	15
H_2_O_2_-20	20	75	10	15
H_2_O_2_-30	30	75	10	15

**Table 5:** Contact angle and surface free energy of MAPP-modified GFRP/WPC.

Sample ID	Distilled Water Contact Angle (°)	Ethylene Glycol Contact Angle (°)	Surface Free Energy ($mJ/m^{2}$)	Polar Component ($mJ/m^{2}$)	Dispersive Component ($mJ/m^{2}$)
Control	99.84	72.06	27.902	0.548	27.354
MAPP-1	98.84	70.65	28.719	0.609	28.110
MAPP-3	99.12	71.97	27.135	0.746	26.389
MAPP-5	98.93	71.60	27.423	0.742	26.681
MAPP-7	99.32	71.95	27.400	0.682	26.718

**Table 6:** Thermal stability characteristics and crystallinity of MAPP-modified GFRP/WPC.

Sample ID	$T_{5wt\%}$ (°C)	$T_{max}$ (°C)	$R_{600}$ (%)	$T_{m}$ (°C)	$∆H_{m}$ (J/g)	$T_{c}$ (°C)	$∆H_{c}$ (J/g)	$X_{c}$ (%)
Control	323.83	465.76	9.53	164.7	79.7	121.7	–77.9	50.85
MAPP-1	321.72	461.33	12.08	164.4	73.3	120.7	–76.6	47.39
MAPP-3	321.14	461.26	13.38	164.9	84.1	120.2	–77.2	55.89
MAPP-5	320.60	460.23	13.69	165.3	87.5	120.4	–78.2	59.81
MAPP-7	319.20	461.65	14.45	164.8	78.5	120.4	–73.6	55.24

**Table 7:** Contact angle and free energy of H_2_O_2_-modified GFRP/WPC.

Sample ID	Distilled Water Contact Angle (°)	Ethylene Glycol Contact Angle (°)	Surface Free Energy ($mJ/m^{2}$)	Polar Component ($mJ/m^{2}$)	Dispersive Component ($mJ/m^{2}$)
Control	99.84	72.06	27.902	0.548	27.354
H_2_O_2_-5	100.22	73.10	26.852	0.604	26.248
H_2_O_2_-10	109.54	73.05	43.256	0.869	42.387
H_2_O_2_-15	110.20	75.44	39.342	0.634	38.708
H_2_O_2_-20	111.30	76.68	38.909	0.737	38.127
H_2_O_2_-30	108.14	72.24	42.016	0.587	41.428

**Table 8:** Thermal stability properties and crystallinity of H2O2-modified GFRP/WPC.

Sample ID	$T_{5wt\%}$ (°C)	$T_{max}$ (°C)	$R_{600}$ (%)	$T_{m}$ (°C)	$∆H_{m}$ (J/g)	$T_{c}$ (°C)	$∆H_{c}$ (J/g)	$X_{c}$ (%)
Control	323.83	465.76	9.53	164.7	79.7	121.7	–77.9	50.85
H_2_O_2_-5	331.21	460.35	10.21	164.8	81.5	120.2	–81.5	51.99
H_2_O_2_-10	332.46	460.33	13.29	164.8	82.4	120.4	–75.2	52.57
H_2_O_2_-15	329.84	462.01	13.48	164.6	87.5	120.6	–74.9	55.82
H_2_O_2_-20	331.40	462.58	16.71	164.4	78.4	120.4	–74.0	50.02
H_2_O_2_-30	323.05	461.86	8.60	165.1	86.3	119.6	–84.9	55.06

## Data Availability

The original contributions presented in this study are included in the article. Further inquiries can be directed to the corresponding author.
